# The Effectiveness of Teaching Applied Transference Focused Psychotherapy on the Attitudes and Technical Confidence of Psychiatry Trainees in Malaysia, on the Management of Patients with Personality Disorder.

**DOI:** 10.1192/bjo.2025.10250

**Published:** 2025-06-20

**Authors:** Muhammad Nur Arif Abdul Rassip, Salina Mohamed, Salmi Razali, Dinah Rasidi, Tennyson Lee

**Affiliations:** 1 Deancross Personality Disorder Service, East London NHS Foundation Trust, London, United Kingdom; 2 Department of Psychiatry, Universiti Teknologi MARA (UiTM), Puncak Alam, Malaysia; 3 Centre for Understanding Personality, London, United Kingdom

## Abstract

**Aims:** Psychiatric trainees are often the “first responders” to manage patients with personality disorders but receive little specific training in this. Transference Focused Psychotherapy (TFP) is a manualized evidence-based treatment for severe personality disorders based on a psychodynamic approach that focuses on object relations theory. There is an expanding experience in applying TFP in different psychiatric settings. While there is evidence of effective training of applied TFP in the UK, Europe, South Africa and India, TFP training in Malaysia is a relatively new concept.

The aim of the study was to evaluate the effectiveness of a series of teaching sessions on TFP as applied to patients with personality disorders, on improving the attitude and technical confidence of psychiatric trainees in one of the major universities in Malaysia (UiTM), in their clinical encounters with patients with personality disorders.

**Methods:** A cohort of psychiatric trainees at UiTM received four 2-hour teaching sessions on applied TFP over consecutive weeks via video teleconference. Nineteen trainees completed 2 questionnaires, pre and post four teaching sessions. The cohort included first-year trainees (n=7) and senior trainees (n=12, comprising second, third and final-year psychiatry trainees). The questionnaires used were the Attitude to Personality Disorder Questionnaire (APDQ) and the Clinical Confidence with Personality Disorder Questionnaire (CCPDQ), both are validated instruments with good psychometric properties.

**Results:** The mean age of participants was 33.18 years, with an average of 2–8 years of experience in psychiatry. Quantitative analyses revealed significant improvements in both attitudes and clinical confidence. For the Attitudes to Personality Disorder Questionnaire (APDQ), the mean pre-intervention score was 71.11±9.87, while the post-intervention mean score was 65.05±10.79, representing a statistically significant reduction (t(18) = 4.11, p=0.00065). This finding indicates improved attitudes toward patients with personality disorders. Similarly, the Clinical Confidence with Personality Disorder Questionnaire (CCPDQ) showed a significant increase in confidence levels. The mean pre-intervention score was 16.11±6.38, which rose to 22.58±7.76 post-intervention (t(18) = −3.58, p=0.0021).

**Conclusion:** Teaching sessions on applied TFP to personality disorders can significantly improve psychiatric trainees’ attitudes and technical confidence in clinical encounters with patients with personality disorders. Given the low resource requirements (8 hours of training, delivered remotely), and the growing international experience of effective teaching of applied TFP training, it may be considered not only in Malaysia but in a range of countries.

